# Extrapyramidal symptoms after exposure to calcium channel blocker-flunarizine or cinnarizine

**DOI:** 10.1007/s00228-017-2247-x

**Published:** 2017-04-06

**Authors:** Kai-Ming Jhang, Jing-Yang Huang, Oswald Ndi Nfor, Yu-Chun Tung, Wen-Yuan Ku, Chun-Te Lee, Yung-Po Liaw

**Affiliations:** 1 Department of Public Health and Institute of Public Health, Chung Shan Medical University, No. 110, Sec. 1 Jianguo N. Rd., Taichung City, 40201 Taiwan; 2 Department of Neurology, Lu-Tung Christian Hospital, Changhua, Taiwan; 3 Department of Pharmacy, Taichung Veterans General Hospital, Taichung, Taiwan; 4 Department of Psychiatry, Chung Shan Medical University Hospital, Taichung, Taiwan; 5 Department of Family and Community Medicine, Chung Shan Medical University Hospital, Taichung, Taiwan

**Keywords:** Cinnarizine, Flunarizine, Calcium channel blocker, National Health Insurance Research Database, Taiwan, Extrapyramidal symptom, Movement disorder

## Abstract

**Purpose:**

Flunarizine (fz) and cinnarizine (cz) have well-known extrapyramidal side effects (EPSEs). The aim of this study was to evaluate the incidence and occurrence time of cz- and fz-related EPSEs.

**Method:**

Patients who took fz or cz for more than 1 month were identified from the longitudinal health insurance database 2005 and 2010. Excluded were patients with any of the underlying diseases that may cause parkinsonism. Drug-induced EPSEs were defined as the new diagnosis of parkinsonism, dyskinesia, or secondary dystonia during drug use or within 3 months after discontinuing the medication. Age- and sex-matched controls were included in this study.

**Results:**

Recruited for analysis were individuals who took fz (*n* = 26,133) and cz (*n* = 7186). The incidence rates of fz- and cz-induced EPSEs were 21.03 and 10.3 per 10,000 person-months, respectively. The hazard ratios (HRs) of EPSEs among fz and cz subjects were 8.03 (95% CI 6.55–9.84) and 3.41 (95% CI 2.50–4.63) when compared with the control individuals. Both fz and cz patients had a higher cumulative incidence of EPSEs than their control individuals (*p* < 0.001). Among subjects who took fz, the incidence of EPSEs was higher in the second than first year of drug exposure (45.59 vs 21.03 per 10,000 person-months).

**Conclusions:**

Fz and cz significantly increased the risk of parkinsonism, dyskinesia, and dystonia. Potential benefits and risks should be weighed when considering long-term use of these drugs especially fz.

**Electronic supplementary material:**

The online version of this article (doi:10.1007/s00228-017-2247-x) contains supplementary material, which is available to authorized users.

## Introduction

EPSEs are drug-induced movement disorders. These symptoms include dystonia (continuous spasms and muscle contractions), akathisia (motor restlessness), parkinsonism (characteristic symptoms such as rigidity and slowness of movement), tremor, and tardive dyskinesia (irregular, jerky movements). Most patients with drug-induced EPSEs are associated with lipophilic dopamine D2 receptor blockers prescribed for psychotic disorders and depression [1]. The first case of fz-induced parkinsonism was reported in 1984 [2]. From that moment, many related cases have been reported [3–9]. Fz and cz are both calcium channel blockers with a similar structure and pharmacologic effects. Fz is a di-fluorinated derivative of cz and has a 2.5–15 time stronger potency than cz [10]. Other than the calcium entry blocking effect, fz and cz also have antihistaminic, antiserotoninergic, and antidopaminergic activities. Because of the D2 receptor blocking activity [11], both fz and cz have EPSEs including parkinsonism, orobuccolingual dyskinesia, dystonia, and akathisia [5, 12]. Previous case series showed that elderly woman and history of essential tremor may probably be risk factors for the development of EPSE following cz or fz exposure [4, 5].

Until now, only few studies have used a large sample size to investigate fz- and cz-induced extrapyramidal symptoms. In Taiwan, fz and cz are frequently prescribed for vertigo, migraine prophylaxis, and cerebrovascular blood flow insufficiency. The aim of this study was to investigate the incidence and occurrence time of cz- and fz-related EPSEs.

## Materials and methods

### Data source

This retrospective cohort study used data from the Longitudinal Health Insurance Databases (LHIDs) 2005 and 2010. Both databases contain registration data and medical claims of 1,000,000 individuals randomly sampled from the 23.68 million beneficiaries registered in the National Health Insurance Research Database (NHIRD). The NHIRD contains a comprehensive health care information including diagnoses, prescriptions, and information about inpatient and outpatient care during 1996–2011. It covers over 99% of the 23 million residents in Taiwan. This study was approved by the Institutional Review Board of Chung-Shan Medical University Hospital in Taiwan.

### Patient identification

Included in this study were patients who were prescribed cz or fz from 2002 to 2011 for more than 1 month. Patients who had been prescribed cz or fz in 2001 were excluded. A total of 68,924 patients who took either fz (*n* = 54,052), cz (*n* = 13,027), or both (*n* = 1665) were identified. The first prescription date of either cz or fz medication was defined as the index date. Excluded from the study were 10,961 patients who were diagnosed with one or more of the several diseases mentioned below before the index date. They included dementia (International Classification of Diseases, 9th revision, clinical modification (ICD-9-CM) 290.0∼290.43), neurodegeneration (ICD-9-CM 333.0, 333.4, 334.0–334.9), hydrocephalus (ICD-9-CM 331), subdural hemorrhage (ICD-9-CM 432.1), brain tumor (ICD-9-CM 191), Wilson’s disease (ICD-9-CM 275.1), hypoparathyroidism (ICD-9-CM 252.1, 252.8, 252.9, 275.49A), pantothenate kinase-associated neurodegeneration (ICD-9-CM 277.9I), human immunodeficiency virus infection (ICD-9-CM 042, 079.53, 795.71), neurosyphilis (ICD-9-CM 094.89, 094.9), progressive multifocal leukoencephalopathy (ICD-9-CM 046.3), toxoplasmosis (ICD-9-CM 130.0, 130.7), and stroke (ICD-9-CM 431, 432.9, 434, 436). Also excluded were 12,297 patients below the age of 45 and 5728 patients diagnosed with parkinsonism, dyskinesia, or dystonia (ICD-9-CM 332.0, 332.1, 333.90, 333.99, 333.7, and 333.8) before the index date. In addition, patients who used fz and cz simultaneously (*n* = 1016) were excluded.

### Definition of outcomes

Drug-related EPSEs was defined as a diagnosis of parkinsonism, dyskinesia, or symptomatic dystonia (ICD-9-CM 332.0, 332.1, 333.90, 333.99, 333.7, and 333.8) from the index date to 3 months after stopping either fz or cz [8].

To determine the hazard ratio of extrapyramidal symptoms, a control group matched by sex, age, and duration of follow-up was included in the analysis (exposure: control group = 1:2). Control subjects were excluded due to fulfillment of the exclusion criteria listed above. Five thousand six hundred three patients were excluded from the study because there were no suitable control individuals. The potential confounders included sex, age, low income, urbanization, and antipsychotics usage 2 years before the index date (anatomical therapeutic chemical code N05A), co-morbidities (DM, ICD-9-CM: 250), chronic kidney disease (CKD, ICD-9-CM: 585), severe liver dysfunction (ICD-9-CM: 572.2, 571.5, 572.2–572.4), history of essential tremor (ICD-9-CM: 333.1), history of other movement disorders (ICD-9-CM: 333.2, 333.3, 333.5, 333.6), and cardiovascular disease (ICD-9-CM: 410–414, 433, 444)).

### Statistical analysis

Data were analyzed using the SAS software. ANOVA was used to compare the mean difference among groups for continuous variables while chi-square test was used for nominal variables. The hazards ratio (HR) and 95% confidence interval (CI) were estimated using the Cox proportional hazard model. A *P* value < 0.05 was considered statistically significant.

## Results

**Fig. 1:**
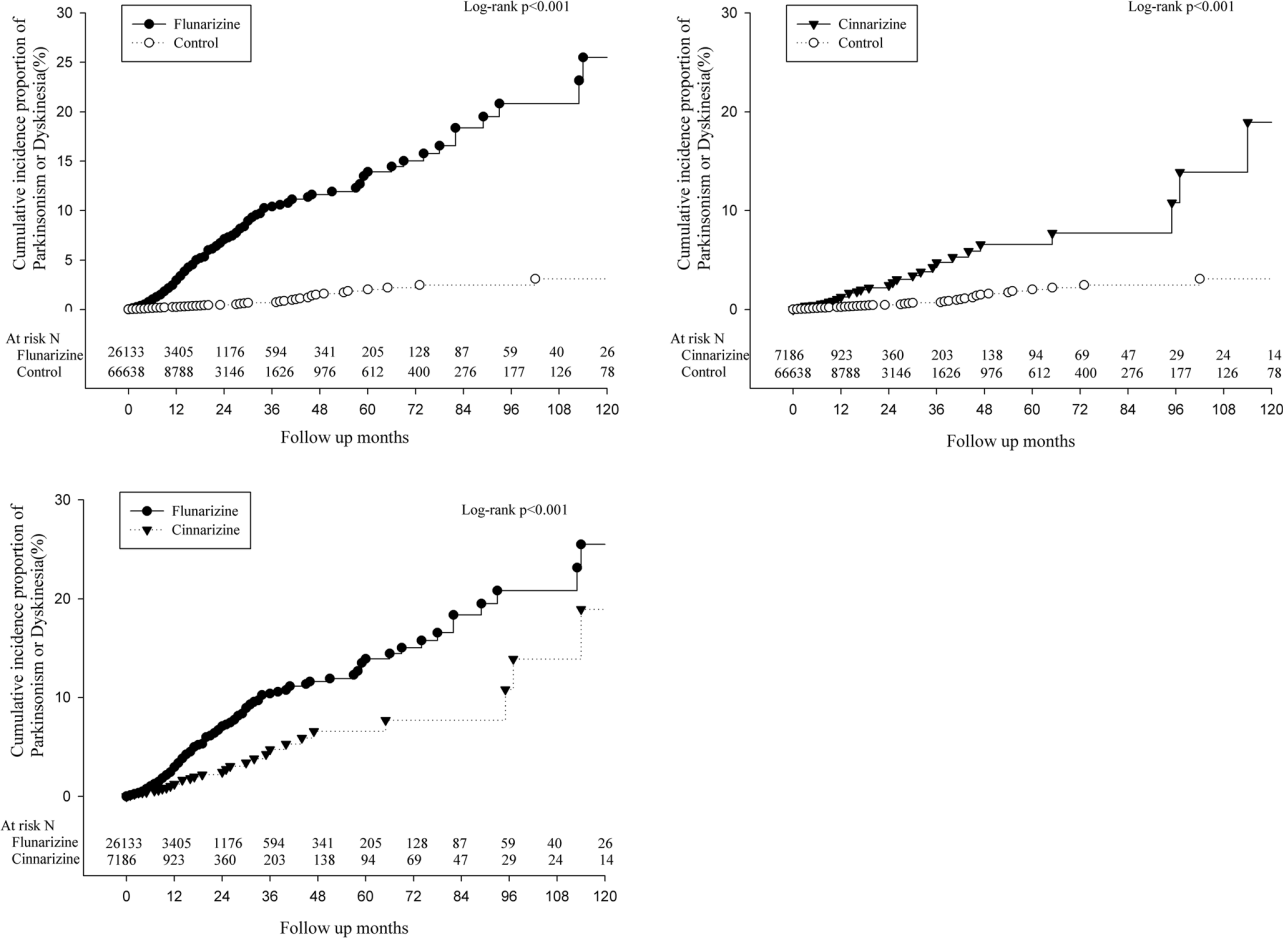
Kaplan-Meier plot of cumulative incidence proportion of parkinsonism or dyskinesia/dystonia by groups

Table 1 shows basic characteristics of study subjects. In the final analysis, 26,133 and 7186 individuals were found to have been prescribed fz or cz, respectively. The matched controls involved 66,638 participants. The incidence rates of fz- and cz-induced EPSEs were 21.03 and 10.3 per 10,000 person-months, respectively (Table 2). Figure 1 shows the cumulative incidence proportion of the EPSEs. Both fz and cz patients had a higher cumulative incidence of EPSEs than their control individuals (Fig. 1 a, b, *p* < 0.001). Patients who were prescribed fz had a higher cumulative incidence than those who were prescribed cz (Fig. 1 c, *p* < 0.001). The incidences of EPSEs in both treatment groups were higher in the second than the first year of exposure (i.e., 45.59 vs 18.62 per 10,000 person-months for fz; 13.89 vs 9.41 per 10,000 person-months for cz). The incidence rates of parkinsonism or dystonia/dyskinesia are listed in supplementary Table 1. Because many patients had antipsychotics or metoclopramide concomitantly, the risk of cz- or fz-related EPSEs might have been overestimated. A sensitivity analysis which excluded patients concomitantly taking antipsychotics, reserpine, or metoclopramide with cz or fz showed that the incidence rates of fz (*n* = 10,020) and cz (*n* = 2567) induced EPSEs were 13.05 (10.74–15.86) and 8.10 (5.04–13.04) per 10,000 person-months.

**Table 1:** Basic characteristics of the study participants

	Flunarizine	Cinnarizine	Control	*p* value
*N*	26,133	7186	66,638	
Initial age	60.94 ± 10.23	62.29 ± 10.16	61.23 ± 10.23	<.0001
Sex				0.56
Male	9449(36.16%)	2549(35.47%)	23,996(36.01%)	
Female	16,684(63.84%)	4637(64.53%)	42,642(63.99%)	
Low income				<.0001
No	25,891(99.07%)	7135(99.29%)	66,231(99.39%)	
Yes	242(0.93%)	51(0.71%)	407(0.61%)	
Urbanization				<.0001
Urban	14,905(57.04%)	4185(58.24%)	41,320(62.01%)	
Normal	8026(30.71%)	1994(27.75%)	18,693(28.05%)	
Rural	3202(12.25%)	1007(14.01%)	6625(9.94%)	
Comorbidity at baseline				
CKD	584(2.23%)	180(2.5%)	1075(1.61%)	<.0001
Severe liver dysfunction	424(1.62%)	107(1.49%)	766(1.15%)	<.0001
Essential tremor history	139(0.53%)	40(0.56%)	131(0.20%)	<.0001
Other movement disorder	64(0.24%)	19(0.26%)	56(0.08%)	<.0001
DM	5888(22.53%)	1738(24.19%)	9936(14.91%)	<.0001
CVD	7419(28.39%)	1985(27.62%)	9347(14.03%)	<.0001
Medication used before baseline				
Antipsychotic	2411(9.23%)	541(7.53%)	1796(2.7%)	<.0001

*N* number, *CKD* chronic kidney disease, *DM* diabetes mellitus, *CVD* cardiovascular disease

**Table 2:** Incidence rate of parkinsonism, dyskinesia, or dystonia in the exposed and control subjects

	Flunarizine		Cinnarizine		Control	
	Case	Incidence rate (per 10,000 person months)	Case	Incidence rate (per 10,000 person months)	Case	Incidence rate (per 10,000 person months)
All period	436	21.03 (19.15–23.1)	61	10.30 (8.02–13.24)	127	2.36 (1.99–2.81)
Within 1 year	301	18.62 (16.63–20.85)	41	9.41 (6.93–12.78)	92	2.23 (1.82–2.74)
From 1 to 2 years	102	45.59 (37.55–55.35)	9	13.89 (7.23–26.69)	13	2.21 (1.28–3.80)

Table 3 shows the HRs for parkinsonism, dyskinesia, or dystonia after exposure to drugs. After adjusting for age, sex, income, urbanization, baseline antipsychotic use, and other comorbidities, the HRs for EPSEs among patients who were exposed to fz and cz were 8.03 (95% CI 6.55–9.84) and 3.41 (95% CI 2.50–4.63), respectively. Table 4 shows the HRs for parkinsonism, dyskinesia, or dystonia for different periods. The risk for EPSEs among subjects who took fz was significantly higher in the second (HR = 18.55, 95% CI 10.32–33.35) than the first year (HR = 7.82, 95% CI 6.17–9.93).

**Table 3:** Cox regression analysis to estimate the hazard ratios of parkinsonism, dyskinesia, or dystonia

	HR	95% C.I.	*p* value
Intervention (ref: control)			
Flunarizine	8.03	6.55–9.84	<.0001
Cinnarizine	3.41	2.50–4.63	<.0001
Sex (ref: Female)			
Male	0.94	0.79–1.10	0.42
Age (per 1 year)	1.06	1.05–1.07	<.0001
Low income (ref: No)			
Yes	1.67	0.92–3.03	0.094
Urbanization (ref: Urban)			
Normal	1.11	0.94–1.32	0.219
Rural	0.88	0.69–1.13	0.31
Comorbidity at baseline (ref: Without)			
CKD	1.31	0.87–1.98	0.20
Severe liver dysfunction	0.40	0.15–1.08	0.071
Essential tremor history	6.14	3.91–9.62	<.0001
Other movement disorders	3.97	1.64–9.58	0.002
DM	1.14	0.95–1.36	0.16
CVD	1.33	1.13–1.58	0.0008
Medication used before baseline (ref: No)			
Antipsychotic	1.82	1.44–2.31	<.0001

*CKD* chronic kidney disease, *DM* diabetes mellitus, *CVD* cardiovascular disease

**Table 4:** Cox regression model to estimate the hazard ratios of parkinsonism, dyskinesia, or dystonia by groups and observation period

	Flunarizine		Cinnarizine	
	HR^a^ (95% C.I.)	*p* value	HR^a^ (95% C.I.)	*p* value
All period (*n* = 99,957)	8.03 (6.55–9.84)	<.0001	3.41 (2.50–4.63)	<.0001
Within 1 year (*n* = 99,957)	7.82 (6.17–9.93)	<.0001	3.45 (2.38–5.00)	<.0001
From 1 to 2 years (*N* = 13,116)	18.55 (10.32–33.35)	<.0001	4.95 (2.11–11.62)	0.0002

Adjusted for age, sex, low-income household, urbanization, comorbidities (chronic kidney disease, severe liver dysfunction, history of essential tremor or other movement disorder, and cardiovascular disease), and antipsychotic use at baseline

*HR* hazard ratio

^a^Reference group is control group

## Discussion

To our knowledge, there are few epidemiologic studies focusing on fz- and cz-induced EPSEs. The incidence rates of EPSEs in patients who took fz and cz were 21.03 and 10.3 per 10,000 person-months, respectively. Patients who took fz and cz were 8.03 and 3.41 times more likely to develop these symptoms. Previously, Fabinai et al. created a point prevalence analysis including 26 patients and found that 50% of chronic fz and cz users had EPSEs [12].

According to reports from Negrotti [8] and Martí-Masso [5] et al., the diagnosis of fz or cz induced parkinsonism should exclude other causes including other offending drugs. For this reason, we performed a sensitivity analysis which excluded patients concomitantly taking antipsychotics, reserpine, or metoclopramide with cz or fz. The incidence rates of fz- and cz-induced EPSEs were 13.05 and 8.10 per 10,000 person-months. The risks for EPSEs were higher among subjects who took fz (HR = 10.59, 95% CI = 7.36–15.22) and cz (HR = 5.49, 95% CI = 3.11–9.70) than their matched control individuals. We did not use the population of sensitivity analysis as our main result because of the following reasons. First, the NHIRD does not contain clinical data; therefore, we could not clarify distinctly which drug was the major cause of EPSEs. More than half of the patients were concomitantly treated with either antipsychotics or metoclopramide and fz or cz. Further investigations are required to understand the interactions between these drugs. Second, the control group may have selection bias when restricting all these medications. To avoid such biases and to eliminate the influence of antipsychotics, adjustments were made for baseline antipsychotic use.

In this study, the fz users had a higher risk of developing EPSEs than the cz individuals. Fz and cz both block striatal dopamine D2 receptors at clinically used doses [11]. Loss of tyrosine hydroxylase in monoaminergic presynaptic neuron leading to dopamine deletion might also be responsible for these side effects [4, 5, 8]. Since fz is 2.5 to 10 times more potent than cz [10], it is no surprise that the risk of EPSEs was 2 to 3 times higher in subjects who took fz.

The incidence of EPSEs 1–2 years after fz prescription (45.59 per 10,000 person-months) was significantly higher than that within the first year (18.62 per 10,000 person-months). The HR increased from 7.82 (95% CI 6.17–9.93) to 18.55 (95% CI 10.32–33.35) among subjects who took fz for more than 1 year. However, this was not obvious in cz individuals. In Fig. 1, the risk of fz-induced extrapyramidal side effects rose rapidly 2–3 years after the drug exposure. Previous literatures have reported a positive relationship between the duration of fz or cz use and the onset of parkinsonism [12, 13]. The present study has strengthened this concept and has also demonstrated the differences that exist between fz and cz. Potential benefits and risks should be weighed when considering long-term use of these calcium channel blockers.

This study has several limitations. First, the NHIRD does not contain information about clinical examination. This study design may have included patients with clinical presentations of parkinsonism who have never sought medical advice. In addition, some authors included clinical improvement after drug withdrawal as one of the diagnostic criteria of DIP [5]. However, such information was not also available in the NHIRD. However, more researchers found that patients did not fully recover after being exposed to cz and fz [8, 14]. Diseases that may cause parkinsonism or dystonia were excluded from this study. Adjustments were made for possible confounders such as antipsychotic use. Second, patients with a diagnosis of Parkinson’s disease or Parkinson plus syndrome might have been included in the analysis since ICD-9-CM codes 332.0 and 332.1 were considered as outcomes. However, according to previous studies, it is hard to distinguish between fz- or cz-induced parkinsonism and idiopathic Parkinson’s disease (IPD) based on clinical presentations [3–6, 8]. One report showed that 43% patients with fz- or cz-induced parkinsonism had a clinical pattern similar to IPD patients. Dopaminergic treatment was also effective in patients with fz- or cz-induced parkinsonism [15]. Furthermore, more evidence supports that patients with fz- or cz-induced parkinsonism were not fully recovered after stopping medications. In addition, some ultimately developed IPD [5, 8]. In this study, ICD-9-CM codes for Parkinson’s disease were included into analysis considering that fz- or cz-induced parkinsonism may be misclassified as IPD. Third, the incidence of akathisia, one of the EPSEs induced by fz or cz [12, 14] could not be obtained. There is no specific ICD-9-CM code for akathisia. Most patients with akathisia had concomitant parkinsonian symptoms [12]; therefore, the incidence could not have been underestimated in this study.

In conclusion, cz and fz significantly increased the risk of extrapyramidal symptoms. Potential risks should be weighed when considering long-term use of these drugs.

## Electronic supplementary material

ESM 1 (DOCX 24 kb)

## Abbreviations

fz: Flunarizine
cz: Cinnarizine
EPSE: Extrapyramidal side effect
HR: Hazard ratio
DIP: Drug-induced parkinsonism
LHID: Longitudinal Health Insurance Database
NHIRD: National Health Insurance Research Database
ICD-9-CM: International Classification of Diseases, 9th revision, clinical modification
CKD: Chronic kidney disease
CI: Confidence interval
IPD: Idiopathic Parkinson’s disease
