# Supplementary material for: Extrapyramidal symptoms after exposure to calcium channel blocker-flunarizine or cinnarizine
Source: Eur J Clin Pharmacol. 2017 Apr 6;73(7):911–6. doi: 10.1007/s00228-017-2247-x (PMC5486934; doi:10.1007/s00228-017-2247-x)
Supplement: Supplementary file 1 — (DOCX 24 kb) [file 228_2017_2247_MOESM1_ESM.docx]

Supplementary table 1 Incidence rate of parkinsonism and dyskinesia/dystonia by groups and follow-up time period

|  | Flunarizine | Cinnarizine | Control |
| --- | --- | --- | --- |
| All period |  |  |  |
| Follow up person months | 207288 | 59204 | 554136 |
| Parkinsonism or dyskinesia/dystonia |  |  |  |
| Case number | 436 | 61 | 130 |
| Incidence rate (per 10000 person months) | 21.03(19.15-23.1) | 10.3(8.02-13.24) | 2.35(1.98-2.79) |
| Parkinsonism |  |  |  |
| Case number | 413 | 52 | 121 |
| Incidence rate (per 10000 person months) | 19.92(18.09-21.94) | 8.78(6.69-11.53) | 2.18(1.83-2.61) |
| Dyskinesia/dystonia |  |  |  |
| Case number | 25 | 9 | 11 |
| Incidence rate (per 10000 person months) | 1.21(0.81-1.78) | 1.52(0.79-2.92) | 0.2(0.11-0.36) |
| Within 1 year |  |  |  |
| Follow up person months | 161661 | 43572 | 424443 |
| Parkinsonism or dyskinesia/dystonia |  |  |  |
| Case number | 289 | 40 | 93 |
| Incidence rate (per 10000 person months) | 17.88(15.93-20.06) | 9.18(6.73-12.51) | 2.19(1.79-2.69) |
| Parkinsonism |  |  |  |
| Case number | 273 | 35 | 85 |
| Incidence rate (per 10000 person months) | 16.89(15-19.01) | 8.03(5.77-11.19) | 2(1.62-2.48) |
| Dyskinesia/dystonia |  |  |  |
| Case number | 18 | 5 | 10 |
| Incidence rate (per 10000 person months) | 1.11(0.7-1.77) | 1.15(0.48-2.76) | 0.24(0.13-0.44) |
| Between 1-2 year |  |  |  |
| Follow up person months | 184035 | 50053 | 485786 |
| Parkinsonism or dyskinesia/dystonia |  |  |  |
| Case number | 384 | 47 | 106 |
| Incidence rate (per 10000 person months) | 20.87(18.88-23.06) | 9.39(7.05-12.5) | 2.18(1.8-2.64) |
| Parkinsonism |  |  |  |
| Case number | 368 | 42 | 98 |
| Incidence rate (per 10000 person months) | 20.00(18.05-22.15) | 8.39(6.20-11.35) | 2.02(1.65-2.46) |
| Dyskinesia/dystonia |  |  |  |
| Case number | 113 | 12 | 23 |
| Incidence rate (per 10000 person months) | 6.14(5.11-7.38) | 2.40(1.36-4.22) | 0.47(0.31-0.71) |

Table 3 Cox regression model to estimate the hazard ratios of Parkinsonism or dyskinesia/dystonia with potential risk factors

|  | HR | 95% C.I. | p-value |
| --- | --- | --- | --- |
| Intervention (ref: control) |  |  |  |
| Flunarizine | 8.61 | 7.05-10.51 | <.0001 |
| Cinnarizine | 3.57 | 2.63-4.85 | <.0001 |
| Mixed | 8.78 | 5.57-13.83 | <.0001 |
| Sex (ref: Female) |  |  |  |
| Male | 0.91 | 0.77-1.07 | 0.23 |
| Age (per 1 year) | 1.06 | 1.05-1.07 | <.0001 |
| Low income (ref: No) |  |  |  |
| Yes | 1.82 | 1.03-3.23 | 0.04 |
| Urbanization (ref: Urban) |  |  |  |
| Normal | 1.13 | 0.95-1.34 | 0.16 |
| Rural | 0.88 | 0.69-1.13 | 0.31 |
| Comorbidity at baseline (ref: Without) |  |  |  |
| CKD | 1.35 | 0.90-2.05 | 0.15 |
| Severe liver dysfunction | 0.41 | 0.15-1.08 | 0.07 |
| Essential tremor history | 6.14 | 3.92-9.62 | <.0001 |
| Other movement disorder | 3.91 | 1.62-9.44 | 0.0024 |
| DM | 1.11 | 0.93-1.33 | 0.24 |
| CVD | 1.34 | 1.14-1.58 | 0.0005 |

CKD: chronic kidney disease; DM: diabetes mellitus; CVD: cardiovascular disease

Table 4. Cox regression model to estimate the hazard ratios of Parkinsonism or dyskinesia/dystonia by follow-up periods

|  | Flunarizine | | Cinnarizine | | Mixed | |
| --- | --- | --- | --- | --- | --- | --- |
|  | HR* (95% C.I.) | p-value | HR* (95% C.I.) | p-value | HR* (95% C.I.) | p-value |
| Parkinsonism or dyskinesia |  |  |  |  |  |  |
| All period | 8.61(7.05-10.51) | <.0001 | 3.57(2.63-4.85) | <.0001 | 8.78(5.57-13.83) | <.0001 |
| Within 1 year | 7.82(6.16-9.92) | <.0001 | 3.50(2.41-5.08) | <.0001 | 7.75(4.33-13.88) | <.0001 |
| Within 2 year | 9.14(7.34-11.37) | <.0001 | 3.55(2.51-5.01) | <.0001 | 9.26(5.67-15.13) | <.0001 |
| Parkinsonism |  |  |  |  |  |  |
| All period | 8.81(7.17-10.83) | <.0001 | 3.25(2.35-4.51) | <.0001 | 8.48(5.27-13.64) | <.0001 |
| Within 1 year | 8.12(6.34-10.40) | <.0001 | 3.32(2.23-4.93) | <.0001 | 7.74(4.22-14.19) | <.0001 |
| Within 2 year | 9.50(7.58-11.92) | <.0001 | 3.40(2.37-4.89) | <.0001 | 9.36(5.65-15.52) | <.0001 |
| Dyskinesia |  |  |  |  |  |  |
| All period | 5.56(2.70-11.46) | <.0001 | 6.82(2.78-16.72) | <.0001 | 11.37(2.50-51.68) | 0.002 |
| Within 1 year | 4.47(2.03-9.85) | 0.0002 | 4.70(1.59-13.90) | 0.005 | 6.71(0.85-52.79) | 0.07 |
| Within 2 year | 12.41(7.86-19.58) | <.0001 | 4.19(2.08-8.45) | <.0001 | 14.62(6.24-34.22) | <.0001 |

Adjusted for age, sex, low income household, urbanization, comorbidities at baseline

*Reference group is Control group
